# Could sample variance be responsible for the parity-violating signal seen in the Baryon Oscillation Spectroscopic Survey?

**DOI:** 10.1098/rsta.2024.0034

**Published:** 2025-02-13

**Authors:** O. H. E. Philcox, J. Ereza

**Affiliations:** ^1^ Department of Physics, Columbia University, New York, NY 10027, USA; ^2^ Simons Society of Fellows, Simons Foundation, New York, NY 10010, USA; ^3^ Department of Physics, Stanford University, Stanford, California 94305, USA; ^4^ Instituto de Astrofísica de Andalucía (CSIC), Glorieta de la Astronomía, Granada E-18080, Spain

**Keywords:** large-scale structure, correlation functions, inflation, simulations

## Abstract

Recent works have uncovered an excess signal in the parity-odd four-point correlation function measured from the Baryon Oscillation Spectroscopic Survey (BOSS) galaxy catalogue. If physical in origin, this could indicate new parity-breaking processes in inflation. At heart, these studies compare the observed four-point correlator with the distribution obtained from parity-conserving mock galaxy surveys; if the simulations underestimate the covariance of the data, noise fluctuations may be misinterpreted as a signal. To test this, we reanalyse the BOSS CMASS parity-odd dataset with the noise distribution model using the newly developed GLAM-Uchuu suite of mocks. These comprise full N-body simulations that follow the evolution of $2000^{3}$ dark matter particles and represent a significant upgrade compared with the formerly used MultiDark-Patchy mocks, which were based on an alternative (non N-body) gravity solver. We find no significant evidence for parity-violation (with a baseline detection significance of 1.0σ), suggesting that the former signal (2.9σ with our data cuts) could be caused by an underestimation of the covariance in MultiDark-Patchy. The significant differences between results obtained with the two sets of BOSS-calibrated galaxy catalogues (whose covariances differ at the 10−20% level) showcase the heightened sensitivity of beyond-two-point analyses to nonlinear effects and indicate that previous constraints may suffer from large systematic uncertainties.

This article is part of the discussion meeting issue ‘Challenging the standard cosmological model’.

## 1. Introduction

Is the large-scale Universe invariant under a parity transformation? According to the standard cosmological paradigm, the answer is yes, but a slew of recent observations have challenged this notion. In particular, several works have obtained evidence for *birefringence* in the *Planck* cosmic microwave background (CMB) polarization (e.g. [1–7]), which could indicate novel tensorial physics such as an axion–photon coupling [8]. Moreover, analyses of the four-point correlation function (hereafter 4PCF) from spectroscopic galaxy surveys have found an excess scalar parity-violating signal [9,10] (using methods first discussed in [11]). While it seems unlikely that these effects are related, each could be an intriguing hint of physics beyond the standard model; however, one must keep in mind the possibility that the signatures could be non-physical in origin. In this work, we focus on the galaxy parity-violation measurements, with the hope of disentangling novel phenomena from analysis systematics.

To constrain parity-violation with the distribution of galaxies (which transforms as a scalar), one requires statistics based on the 4PCF or beyond. This occurs since tetrahedra (which describe the vertices of a quadruplet of galaxies) are the first chiral polyhedron, and thus the simplest shape for which one can distinguish reflection and rotation (as discussed in [11], see also [12–16]). Roughly speaking, parity-violation measurements proceed by iterating over all possible tetrahedra of galaxies in a survey and counting the number that are left- and right-handed as a function of shape (which is a 4PCF in disguise).^1^ Efficient methods for computing this exist (e.g. [17,18]; using mathematical tools developed in [19]), which have facilitated parity measurements from the SDSS-III Baryon Oscillation Spectroscopic Survey (BOSS) galaxy catalogue containing $O(10^{6})$ galaxies [20,21].

In essence, the analyses of [9] and [10] proceed by computing the parity-odd 4PCF of BOSS data and interpreting this result in terms of the expected distribution arising from cosmic variance and shot-noise. Both works report significant deviations from the null distribution [9] and find a 2.9σ excess in the CMASS sample, while [10] obtained a 7.1σ (3.1σ) deviation for CMASS (LOWZ) galaxies. These analyses differ in their approach and assumptions [9]; primarily make use of non-parametric rank tests, while [10] utilize $\chi^{2}$ tests, facilitated either by rescaling a theoretical covariance or projecting the data into a low-dimensional subspace; furthermore, they find larger signals when adopting finer radial binning.

Analysis differences notwithstanding, both [9] and [10] find an intriguing excess in the 4PCF; if physical, this would suggest parity-violating processes at work in the Universe, which would be of considerable interest to both the observational and theoretical communities. For the former, we note that searches for parity-violation have also been performed using the CMB four-point function [22,23] (and the tensorial three-point function [24]), and there remains the intriguing possibility that such effects could be searched for in galaxy shape and spin statistics (e.g. [25–30]). To date, none of these studies have reported a significant detection, with the CMB non-detection casting doubt upon the galaxy results, given that many reasonable primordial parity-violating models should show strong signatures therein [22,23]. On the theory side, a wide variety of works have considered parity-violating models both in the scalar and tensor sector (e.g. [14,16,31–51]). Applied to BOSS data [44], drew the general conclusions: (i) late-time solutions (i.e. non-inflationary) are heavily suppressed on large scales and (ii) none of the theoretical models yet analysed are consistent with the observational data.

Perhaps the least exciting explanation for the BOSS parity-odd-excess is a mischaracterization of the 4PCF noise distribution (a conclusion supported by the recent re-analysis of [52]). At the heart of both previous analyses is a $\chi^{2}$-like quantity:


(1.1) $$\begin{array}{r} {\tilde{\chi }}^{2}=\zeta_{odd}^{T}C^{-1}\zeta_{odd}, \end{array}$$

where $\zeta_{\mathrm{odd}}$ is the (possibly compressed) 4PCF and C is some covariance matrix, calibrated to a suite of realistic galaxy catalogues, namely, the MultiDark-Patchy suite [53]. If (i) ζ obeys Gaussian statistics, and (ii) C is the true covariance of ζ, ${\tilde{\chi }}^{2}$ follows a $\chi^{2}$ distribution (with the null theory $\zeta_{\mathrm{odd}}=0$), thus the value of ${\tilde{\chi }}^{2}$ obtained from BOSS can be interpreted as a parity-violation detection probability. Of course, the actual analyses are somewhat more nuanced and take into account effects such as the finite number of mocks used to estimate C and non-Gaussianities in the distribution of ${\tilde{\chi }}^{2}$ (via a simple invocation of simulation-based inference) [9,10]. However, all analyses make a key assumption: *that the noise distribution of $\zeta_{\mathrm{odd}}$ from BOSS matches the noise distribution of $\zeta_{\mathrm{odd}}$ from the* MultiDark-Patchy *mock catalogues*.

In this work, we test the above assumption. This is facilitated by a new suite of BOSS mocks: the GLAM-Uchuu galaxy catalogues described in [54]. These are N-body cosmological simulations that follow the evolution of $2000^{3}$ dark matter particles, each with a particle mass of $1.06\times 10^{10}\;h^{-1}M_{⊙}$. The cosmological parameters adopted are $\Omega_{m,0}=0.309$, $\Omega_{b,0}=0.0486$, $\Omega_{\Lambda ,0}=0.691$, h=0.677, $n_{s}=0.9667$ and $\sigma_{8}=0.816$, representing the best-fit ΛCDM parameters corresponding to the cosmology [55]. By contrast (and due principally to 10 years less of Moore’s law), the previously used MultiDark-Patchy catalogues rely on an approximate model for gravitational evolution, rather than employing N-body codes. The Patchy code generates fields for dark matter density and peculiar velocity on a mesh, utilizing Gaussian fluctuations and implementing the augmented Lagrangian perturbation theory scheme of [56].

While alternative methods to N-body simulations are more computationally efficient, they necessarily sacrifice some accuracy. This implies that the MultiDark-Patchy simulations fail to generate a precise matter density field compared with a full N-body simulation such as GLAM-Uchuu, which fully encodes nonlinear gravitational evolution (up to resolution and baryonic effects). The feasibility of the resulting MultiDark-Patchy galaxy catalogues in a real universe is uncertain, which introduces ambiguity in their covariance error estimates for the BOSS clustering statistics, particularly when they are applied to statistics of much higher order than used for the original validation tests. In addition, the generation of these catalogues involves specifying a significant number of parameters (five), leading to a remarkable degree of parameter degeneracy. By contrast, GLAM-Uchuu has only one free parameter. A further important difference is that, unlike MultiDark-Patchy, GLAM-Uchuu includes the redshift-dependent evolution of galaxy clustering. While the MultiDark-Patchy catalogues were generated using a single $2.5\;h^{-1}\mathrm{Gpc}$ box, the GLAM-Uchuu catalogues were generated by joining boxes of different redshift into spherical shells, reproducing the clustering evolution with redshift (see [54] for a detailed description of this method). Moreover [57], recently reported some evidence of model specification errors in MultiDark-Patchy, and [54] found a notable underestimation in the two-point covariances.

In the below, we perform a similar analysis to [9] but replace MultiDark-Patchy with the GLAM-Uchuu galaxy catalogues. Assuming that they better characterize the null $\zeta_{\mathrm{odd}}$ distribution (which is a fair assumption, viz. the above discussion), this will allow us to test whether the formerly reported BOSS 4PCF excess could be explained by a simple miscalibration of the data’s sample variance.

## 2. Methods

Our observational dataset is the 12th data release (DR12) of the BOSS, part of SDSS-III [20,21,58]. As in [9], we use the high-redshift CMASS sample (0.43<z<0.7) and split the data into the North and South galactic regions (denoted ‘N’ and ‘S’ hereafter). Adopting the standard BOSS systematic weights [59,60] (which we caution have been validated only for the two-point functions), the effective number of galaxies in our sample is 605884 and 225469 for CMASS-N and CMASS-S, respectively.

We use two sets of mock catalogues in this study: MultiDark-Patchy and GLAM-Uchuu. Both model the full BOSS footprint (split across the different observational chunks) with similar redshift distributions and observational masks. Here, we use $N_{\mathrm{sim}}=2042$ MultiDark-Patchy and $N_{\mathrm{sim}}=2000$ GLAM-Uchuu mocks.^2^ Both sets of catalogues include the observational veto mask and estimated fibre collision weights, emulating the observational sample. We additionally utilize random catalogues to characterize the unperturbed galaxy distribution (from the mask and radial distribution). These have been separately generated for each dataset (BOSS, GLAM-Uchuu and MultiDark-Patchy) and have size 40×, 30× and 20× the data, respectively. For the GLAM-Uchuu simulations, the volume of the CMASS-N sample exceeds that of the original simulation volume ($1h^{-3}{\mathrm{Gpc}}^{3}$); as in [54], we account for this in covariances by performing the (approximate) rescaling $C_{glam}\to (V_{sim}/V_{data})C_{glam}$.

**Figure 1.**
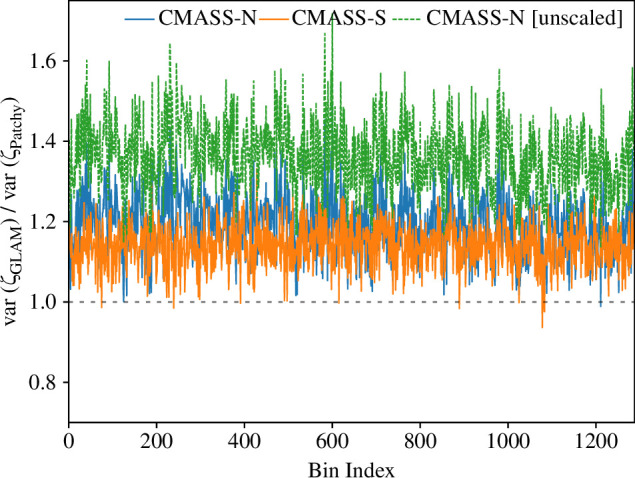
Comparison of the parity-odd 4PCF variance estimated with two suites of galaxy mock catalogues: GLAM-Uchuu (N-body) and MultiDark-Patchy (mesh, with augmented Lagrangian perturbation theory). We show results for the two BOSS samples, as indicated in the caption, and collapse all 1288 4PCF bins into one dimension. For CMASS-N, we rescale the GLAM-Uchuu covariance by the ratio of simulation to data volumes [54]; the unscaled results are shown in green and are clearly discrepant. In both samples, the GLAM-Uchuu variance exceeds that of MultiDark-Patchy by 10–20%.

Given the datasets, 4PCFs are obtained using the encore code [17,61]. This computes the following quantity (with a complexity quadratic in the number of galaxies):


(2.1) $$\begin{array}{rl} \zeta_{\ell_{1}\ell_{2}\ell_{3}}(r_{1},r_{2},r_{3})= & \int dx\;d{\hat{r}}_{1}d{\hat{r}}_{2}d{\hat{r}}_{3}P_{\ell_{1}\ell_{2}\ell_{3}}^{*}({\hat{r}}_{1},{\hat{r}}_{2},{\hat{r}}_{3})\\ & \times \;\delta (x)\delta (x+r_{1})\delta (x+r_{2})\delta (x+r_{3}), \end{array}$$

where δ is the galaxy overdensity,^3^ and $P_{\ell_{1}\ell_{2}\ell_{3}}$ is an angular basis function relative to one vertex of the galaxy tetrahedron (as defined in [19]; see also the BiPoSH basis [63]). The $r_{i}$ parameters give the (discretized) tetrahedron side lengths, while $\ell_{i}$ index the Fourier complement of the internal angle, with odd $\ell_{1}+\ell_{2}+\ell_{3}$ encoding parity-violation. Our binning follows [9]; we include all ℓ up to ℓ=5, but drop ℓ=5 due to residual leakage from the window function, and use 10 radial bins with $r_{i}\in [20,160)h^{-1}\mathrm{Mpc}$, additionally dropping any configurations with internal radii (i.e. the three tetrahedron sides not specified by $r_{1,2,3}$) below $14h^{-1}\mathrm{Mpc}$, through bounds on $|r_{i}-r_{j}|$. Figure 1 shows a comparison between the 4PCF variances obtained in MultiDark-Patchy and GLAM-Uchuu; the implications of this will be discussed below.

**Table 1.** Probability of a detection of parity-violation from the BOSS dataset, obtained using the rank test shown in figures 2 and 3. We give the one-tailed probability of BOSS compared with the distribution of GLAM-Uchuu (left columns, new to this work) and MultiDark-Patchy (right columns, obtained also in previous work) simulations, and present also the effective Gaussian significances. The second column gives the effective volume of each sample in $h^{-3}{Gpc}^{3}$ units. The analyses utilizing the GLAM-Uchuu suite find no detection of parity-violation.

sample	volume	GLAM-Uchuu	MultiDark-Patchy
combined	1.94	69.5% 1.0σ	99.6% 2.9σ
CMASS-N	1.15	67.2% 1.0σ	98.8% 2.5σ
CMASS-S	0.43	62.2% 0.9σ	97.3% 2.2σ

**Figure 2.**
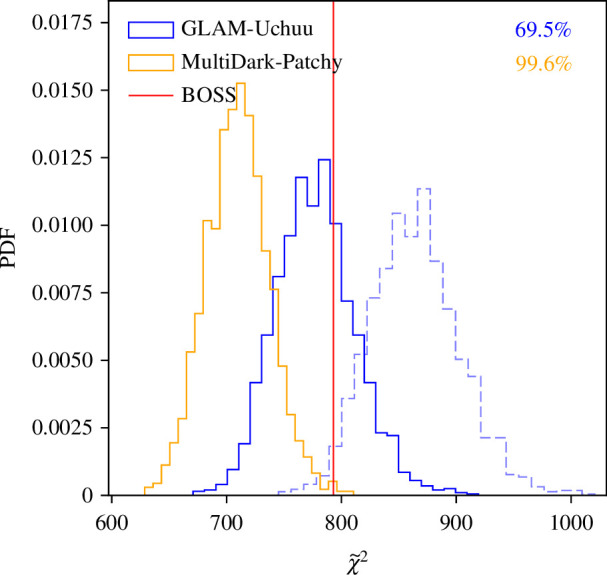
Distribution of the parity-odd statistic ${\tilde{\chi }}^{2}$ (1.1) from 2042 MultiDark-Patchy catalogues (used in previous analyses) and 2000 GLAM-Uchuu catalogues (new to this work), with the BOSS result shown as a vertical red line. We show results from the combined CMASS observational dataset, with those from the two subsamples displayed in figure 3. The rank-test probabilities (i.e. detection significances) are shown in the upper right corner, and we note that the normalization is arbitrary. Using the GLAM-Uchuu catalogues, we find no evidence for parity-violation in BOSS, but a strong preference when the 4PCF noise distribution is modelled with the MultiDark-Patchy suite. Furthermore, we find ≥3σ evidence for parity-violation in 28% of the (parity-conserving) GLAM-Uchuu mocks, when analysed with MultiDark-Patchy. Numerical constraints are given in table 1, and dashed lines show the (incorrect) results without applying the GLAM-Uchuu CMASS-N volume rescaling factor.

**Figure 3.**
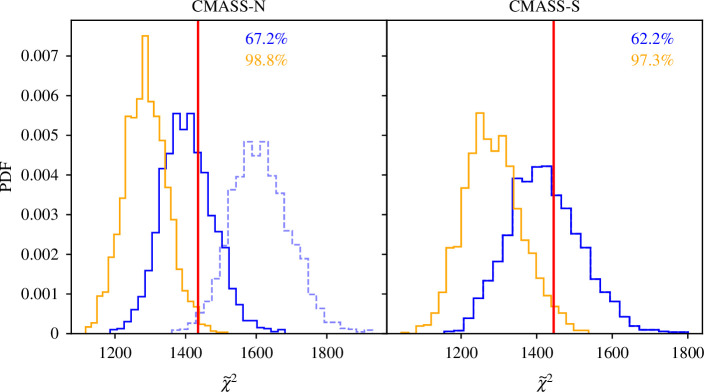
As figure 2, but assessing the probability of parity-violation in each of the BOSS data chunks (indicated by the title). The PDFs of GLAM-Uchuu are significantly shifted to larger values than MultiDark-Patchy (which sets the normalization) in both samples, as expected from figure 1. We find no detection of parity-violation in either sample (see table 1). Without the necessary GLAM-Uchuu volume rescaling factor, CMASS-N shows a strong ‘anti-detection’, as shown in the dashed lines.

**Figure 4.**
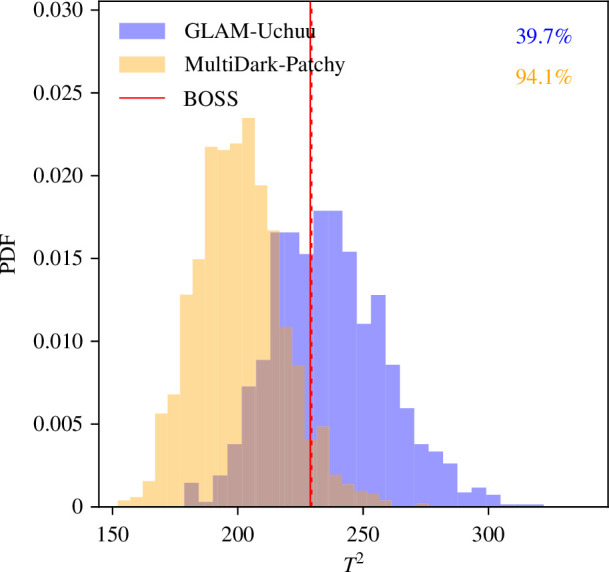
Alternative test for parity-violation, projecting the parity-odd statistic onto the 250 lowest-noise eigenmodes of a fiducial covariance matrix, then computing the distribution of the $T^{2}$ statistic (2.2) using the empirical GLAM-Uchuu or MultiDark-Patchy covariance. PDFs are obtained from jackknifing (histograms) and analytic Gaussian theory (lines) and we show results for the three largest samples combined, with the numbers on the top-right giving the detection probabilities relative to the mocks. The full (dashed) red lines show the BOSS results when analysed using the GLAM-Uchuu (MultiDark-Patchy) mocks. We find no significant detection of parity-violation when using GLAM-Uchuu but a detection when using MultiDark-Patchy.

Computation of the 4PCFs was performed on a high-performance figure 2 computing cluster. Thanks to encore’s efficient C + +and avx implementation, ≈60 min was needed to process each mock (including both data chunks) on a 40-CPU machine, thus the full analysis required ≈30000 CPU-hours.^4^ The full data-vector, $\zeta_{\mathrm{odd}}=\left\{\zeta_{\ell_{1}\ell_{2}\ell_{3}}(r_{1},r_{2},r_{3})\;\forall \;r_{i},\ell_{i}\right\}$, contains $N_{\mathrm{bins}}=1288$ bins for each of the two (assumed independent) data chunks.

To analyse the data, we follow the methods of [9], making use of an analytic covariance, C, computed as described in [64].^5^ This is estimated under various simplifying assumptions (e.g. in the Gaussian limit and without a window), thus we use it only as an approximate tool for dimensionality reduction (noting that former works [9,61,64] have found that it well-represents the empirical correlation structure of the 4PCF, but not the variance). On each of the data and mocks, we estimate a *pseudo*-$\chi^{2}$ statistic (hereafter ${\tilde{\chi }}^{2}$) from the 4PCF and fiducial covariance via (1.1). If C is close to the true covariance, this will provide a near-optimal noise weighting in the Gaussian limit. Given the set of ${\tilde{\chi }}^{2}$ values obtained from a suite of (parity-conserving) mock catalogues, we construct an empirical distribution $p_{\mathrm{sim}}({\tilde{\chi }}^{2})$ and assess the probability-to-exceed of BOSS, i.e. $p_{\mathrm{sim}}({\tilde{\chi }}_{\mathrm{BOSS}}^{2})$. This is practically implemented as a rank test, which is a simple form of simulation-based inference. Since we take the distribution of ${\tilde{\chi }}^{2}$ directly from the catalogues, we do not need to assume Gaussianity nor that C well-approximates the true covariance; however, we must assume that $p_{\mathrm{sim}}({\tilde{\chi }}^{2})$ is accurate, i.e. that the simulated sample variance matches that of the observations. When analysing multiple data chunks, we perform an effective-volume-weighted sum of the ${\tilde{\chi }}^{2}$ values, noting that this again cannot induce bias.^6^

We additionally perform a second test for parity-violation, used in both [9] and [10] (following [61,66]). Given the high dimensionality of $\zeta_{odd}$, we cannot compute the empirical covariance $C_{sim}={\left\langle \zeta_{odd}\zeta_{odd}^{T}\right\rangle }_{sim}$ directly from the mock catalogues. Instead, we use the fiducial covariance C to define a minimum-variance eigenbasis containing $N_{\mathrm{eig}}=250$ elements, into which the data and mock 4PCFs are projected via some $N_{\mathrm{eig}}\times N_{\mathrm{bins}}$ operator Π.^7^ This is analysed with the following statistic:


(2.2) $$\begin{array}{r} T^{2}=(\Pi \zeta_{odd}{)}^{T}C_{sim}^{-1}(\Pi \zeta_{odd}), \end{array}$$

which is closely related to (1.1) but uses the empirical covariance $C_{sim}={\left\langle (\Pi \zeta_{odd})(\Pi \zeta_{odd}{)}^{T}\right\rangle }_{sim}$ estimated from a subset of 800 mocks. Assuming a Gaussian distribution for $\Pi \zeta_{odd}$, (2.2) follows a $T^{2}$ distribution, as discussed in [67]. When combining samples, we add the $T^{2}$ values, weighted as above. In all cases, we evaluate the observed $T^{2}$ values by comparing them with the empirical mock-based distribution, obtained from the remaining $\left(N_{\mathrm{sim}}-800\right)$ simulations. Assuming that the fiducial and empirical covariances differ, this test will provide a different weighting than the rank-based test described above, and could thus differently highlight parity-violating signatures.

## 3. Results

We begin by discussing the empirical 4PCF covariances obtained from the GLAM-Uchuu and MultiDark-Patchy suites. While the correlation structures are very similar (see fig. 3 of [9]), the variances differ considerably, as shown in figure 1. For both samples, the GLAM-Uchuu covariance significantly exceeds that of MultiDark-Patchy, with a roughly uniform rescaling found across the radial and angular 4PCF components (once we have taken into account the GLAM-Uchuu volume rescaling factor described above). The excess covariance reported above is not unsurprising, given that the 4PCF covariance contains contributions from the 2PCF, 3PCF, 4PCF, 5PCF and 8PCF, and it is unclear whether approximate mocks properly include higher-point functions. The above observation is central to the remainder of the paper. If the MultiDark-Patchy 4PCF covariance was underestimated, could this explain the excess parity-odd signal?

In figure 2, we present the main result of this work: a comparison of the parity-violation parameter ${\tilde{\chi }}^{2}$ from BOSS, GLAM-Uchuu and MultiDark-Patchy. This is analogous to fig. 4 of [9] but now includes the empirical distribution from the GLAM-Uchuu catalogues and a more optimal weighting scheme. The results are striking: we find that the BOSS data have larger ${\tilde{\chi }}^{2}$ than 99.6% of the MultiDark-Patchy catalogues but only 69.5% of the GLAM-Uchuu suite. Converted to one-tailed Gaussian significances (cf. table 1), this corresponds to a 2.9σ excess in BOSS when analysed with MultiDark-Patchy (consistent with [9,10] or a 1.0σ deviation using GLAM-Uchuu). If we regard MultiDark-Patchy as the ‘true’ noise distribution, we thus obtain a strong detection of parity-violation from BOSS, but this disappears if we assume GLAM-Uchuu is a more realistic representation of the BOSS survey. Furthermore, we can treat each GLAM-Uchuu mock as a ‘validation’ dataset for the MultiDark-Patchy analysis: in this framework, we find ≥3σ detections of parity-violation in 28% of the GLAM-Uchuu mocks, suggesting significant bias in the MultiDark-Patchy pipeline.

To understand these results further, we show the ${\tilde{\chi }}^{2}$ distributions from each BOSS chunk in turn in figure 3, with associated numerical results given in table 1. In both cases, we find significant differences between the GLAM-Uchuu and MultiDark-Patchy distributions, which occur primarily due to the different 4PCF covariances (cf. figure 1). For both datasets, ${\tilde{\chi }}_{\mathrm{BOSS}}^{2}$ falls close to the centre of the GLAM-Uchuu distribution but in the tails of the MultiDark-Patchy distribution, matching the findings of figure 2.

Finally, we show results from the alternative Gaussian analysis in figure 4. When projecting the combined data onto 250 eigenmodes and comparing with the empirical $T^{2}$ distribution, we find a 1.9σ excess in BOSS when analysed with MultiDark-Patchy or a 0.5σ detection with GLAM-Uchuu. This again indicates no significant evidence for parity-violation in the GLAM-Uchuu analysis.

## 4. Discussion

What do the above results imply for the cosmic parity-violation study of [9]? The precise conclusions depend on our assumptions about the GLAM-Uchuu and MultiDark-Patchy simulation suites. Two choices are apparent:

(1) We assume that the noise distribution of BOSS is well-modelled by the new GLAM-Uchuu catalogues but not by the older MultiDark-Patchy catalogues (which are less realistic, as, for example, they use an alternative method to N-body and do not account for redshift evolution of the galaxy samples). In this case, we find no evidence for parity-violation, with our main analysis yielding only 1.0σ in a one-tail test.
(2) We assume that neither the GLAM-Uchuu nor MultiDark-Patchy suites accurately represent the BOSS noise distribution. In this case, the significant variation in detection significances from two differently produced but similarly calibrated galaxy catalogues indicates that our pipeline may have large systematic errors and cannot be used to claim any strong detection of parity-violation.

In either case, we do not find significant evidence for parity-violation from the BOSS survey (using the approach of [9]), and thus no observational motivation for novel inflationary or late-time physics, in accordance with the latest CMB analyses [22,23] and a recent BOSS reanalysis by [52].

Although the statistical methods differ, this result also has implications for the analysis of [10]. In this work, the main constraints come from an unprojected Gaussian analysis using the theoretical covariance matrices of [64], with the effective volume and shot-noise fitted to the empirical MultiDark-Patchy covariance. Although this introduces mock-based information only through a pair of parameters, one still affects significant variation in the constraints if the GLAM-Uchuu catalogues are replaced with those of MultiDark-Patchy (from figure 1, given the roughly constant variance ratio). The parity-odd excess of [10] is also enhanced if more bins are used in the data-vector; this would be interesting to investigate further with the GLAM-Uchuu catalogues, though we caution that larger dimensionality is often associated with likelihood of non-Gaussianity.

Finally, the analysis described in this work has ramifications for future surveys such as DESI and Euclid [68,69]. These experiments will lead to an explosion in the data volume, facilitating much more precise probes of the parity-violating sector (among many other topics). Enhanced precision does not preclude bias however, and it remains possible that model-independent analyses like the above will be limited by the accuracy of our catalogues, in particular their ability to reproduce the statistical properties of high-dimensional observables. If these hurdles can be surmounted, however, the parity-odd dataset could open doors to a wide variety of exciting inflationary studies.

## Data Availability

The GLAM-Uchuu catalogues are publicly available at Skies and Universes: https://www.skiesanduniverses.org/Products/. The encore code is publicly available at [70].

## Footnotes

^1^
This can be done uniquely for a translationally invariant system; one considers the sign of the triple product $r_{1}\cdot r_{2}\times r_{3}$, where $r_{i}$ is the position vector from the (specified) base to the i-th vertex and we fix $r_{1}<r_{2}<r_{3}$.

^2^
Six MultiDark-Patchy mocks were corrupted in data transfer and are thus dropped from the analysis.

^3^
In practice, we use the data and random catalogues to create a Landy–Szalay-type estimator [62] to remove the window function, as described in [17,61].

^4^
Though the encore GPU implementation described in [17] is considerably faster, we do not use it here due to the far larger number of CPUs available on a typical cluster.

^5^
Specifically, we base our analysis on a fiducial covariance calibrated for the largest data-chunk (CMASS-N), as used in [9]. For each chunk, we rescale the covariance by an effective volume, obtained by fitting C to an empirical covariance obtained from 10% of the MultiDark-Patchy catalogues using a Kullback–Leibler minimization.

^6^
This is motivated by the optimal linear combination of ${\tilde{\chi }}^{2}(z)$ measurements given some assumed signal $\zeta (z)∼b^{4}(z)D^{4}(z)$, noting that both $\mathrm{var}({\tilde{\chi }}^{2})$ and the effective bias b(z)D(z) are approximately independent of redshift [65].

^7^
The choice of $N_{\mathrm{eig}}$ is somewhat arbitrary but [9] found $N_{\mathrm{eig}}=250$ to yield large detection significances with MultiDark-Patchy catalogues while limiting finite-mock effects.
